# The association between serum vitamin A concentrations and virus hepatitis among U.S. adults from the NHANES database: a cross-sectional study

**DOI:** 10.3389/fnut.2024.1387461

**Published:** 2024-08-01

**Authors:** Maoxia Liu, Jianlei Fu, Xuepeng Zhang, Qinyi Fu, Yi Ji, Siyuan Chen

**Affiliations:** ^1^Department of Critical Care Medicine, West China Hospital of Sichuan University, Chengdu, China; ^2^Division of Oncology, Department of Pediatric Surgery, West China Hospital of Sichuan University, Chengdu, China

**Keywords:** serum VA, HBsAg, HCV-RNA, NHANES, virus hepatities

## Abstract

**Objective:**

According to the present study, the relationship between vitamin A (VA) levels and hepatitis virus carriage has been unclear and controversial. This study aimed to determine the potential relationship between serum VA levels and viral hepatitis and to provide ideas for future clinical treatments.

**Methods:**

A cross-sectional study was performed using the 2005–2006 and 2017–2018 National Health and Nutrition Examination Survey (NHANES) datasets. Multiple linear regression and logistic regression were adopted to analyze the association between serological hepatitis B surface antigen (HBsAg) or hepatitis C RNA (HCV-RNA) positivity and VA levels. There were 5,351 HBsAg-related responders and 242 HCV-RNA-related responders, including 52 HBsAg (+) and 104 HCV-RNA (+) responders.

**Results:**

Compared with HBsAg (−) and HCV-RNA (−) respondents, HBsAg (+) and HCV-RNA (+) respondents tended to have lower serum VA levels, respectively [1.63 (1.33 ~ 2.01) vs. 1.92 (1.57 ~ 2.34), *P* < 0.001; 1.54 (1.25 ~ 1.83) vs. 1.78 (1.46 ~ 2.26), *P* < 0.001]. A greater percentage of responders in the subclinical VA deficiency (SVAD) group were HBsAg (+) and HCV-RNA (+) than were those in the normal VA (VAN) group [2.4% (9/374) vs. 0.9% (43/4977), *p* = 0.003; 61.5% (16/26) vs. 40.7% (88/215), *p* = 0.043]. According to the results of the multiple regression analyses of the different models, the serum VA concentration was negatively correlated with HBsAg (+) and HCV-RNA (+) status (*β* = −0.14, 95% CI = −0.30 to −0.01, *p* = 0.066; *β* = −0.29, 95% CI = −0.50 ~ −0.09, *p* = 0.005, respectively). Compared to those with SVAD, patients with VAN were less likely to be serologically HBsAg (+) or HCV-RNA (+) (OR = 0.53, 95% CI = 0.25 ~ 1.10, *p* = 0.089; OR = 0.39, 95% CI = 0.18 ~ 0.84, *p* = 0.016, respectively).

**Conclusion:**

Our study provides evidence that patients who are HBsAg (+) or HCV-RNA (+) have a high incidence of SVAD. Moreover, HBsAg and HCV-RNA positivity are negatively correlated with VA levels, and patients with SVAD are more likely to carry HBsAg (+) or HCV-RNA (+). These findings suggest that the relationship between hepatitis viruses and vitamin A needs to be validated by more basic studies and clinical large-sample randomized controlled trials to provide ideas for new therapeutic targets.

## 1 Introduction

Vitamin A (VA) refers to a group of small fat-soluble molecules composed of a cyclic ring, a polyene side chain, and a polar end group (1). VA is crucial for many important biological functions, including reproduction, embryological development, cellular differentiation, growth, immunity, and vision. VA deficiency (VAD) is common and has been shown to be associated with infection, especially viral infection (2). In our previous study, we observed that most children with hand, foot and mouth disease (HFMD) presented with reduced VA levels, which was associated with their decreased immunity and exacerbation of the disease. The data showed that the reduction of IFN-α and EV71-IgM concentrations was positively correlated with the reduction of VA levels (3). At the same time, our study revealed that VAD was associated with severe sepsis, septic shock and increased PRISM scores (4). Furthermore, VAD may be a marker of death in children with severe sepsis. More than 80% of VA is stored in the liver in the form of retinyl esters (5). The human liver not only functions as a storehouse for VA but also needs retinol for its normal functions. However, whether there is a correlation between VA and hepatitis virus infection is currently unclear.

Retinol is a natural metabolite of VA, and plasma retinol levels indicate VA status (6). The serum retinol concentration, which is positively associated with the dietary retinol concentration, has been shown to have an independent effect on hepatocellular carcinoma (7). According to clinical research, in patients with liver disease, the levels of VA, retinol-binding protein (RBP), and prealbumin (PA) are markedly decreased and are highly significantly correlated with a wide range of concentrations (8, 9). Moreover, in patients with acute hepatitis, as the disease progresses, the plasma concentrations of VA, RBP, and PA increase (8, 10). In contrast, Saron et al. (11) reported lower serum retinol levels in individuals with chronic liver diseases, which was related to the severity of the condition. These findings suggest that VA levels vary among different liver diseases and may be related to disease progression, etiology, and nutritional status. Hepatic stellate cells within liver lobules store approximately 80% of the total body VA in lipid droplets in the cytoplasm (12). These cells also play a pivotal role in the regulation of VA homeostasis (13–15). The burden of liver disease caused by hepatitis B virus (HBV) infection worldwide is great, with approximately 250 million people chronically infected globally (16, 17). Chronic HBV infection is particularly prevalent in Asia, especially in China (18). Piyathilake (19) showed that HBV infection was also a strong predictor of VAD. HBV infection can impact hepatic cells, thereby affecting VA metabolism and storage. The association between VA deficiency and high aflatoxin B1 albumin (AF-ALB) levels may result in impairment of the host immune response, which increases susceptibility to infectious diseases (19).

Hepatitis C virus (HCV) is a human pathogen responsible for acute and chronic liver disease (20) and chronically infects an estimated 71.1 million individuals worldwide (21). In addition to being a simple hepatic viral infection, HCV is now thought to cause metabolic alterations, as much of its life cycle is closely associated with lipid metabolism (22). Several lines of evidence indicate the involvement of HCV in the retinoid pathway (23). VA and other retinoids have recently been demonstrated to inhibit measles virus, EV71 (3) and HCV *in vitro* (24). These findings indicate that HCV infection can affect VA levels, which in turn affects the body’s immunity, further aggravating the patient’s condition.

The aim of this study was to analyze vitamin A levels and hepatitis virus carriage to explore the potential relationship between viral hepatitis and vitamin A and to provide new ideas for clinical treatment.

## 2 Methods

### 2.1 National health and nutrition examination survey

The data analyzed were collected from the 2005–2006 and 2017–2018 NHANES survey cycles.^1^ The NHANES is a nationwide survey conducted annually for the purpose of collecting health and diet information from a representative, non-institutionalized U.S. population. The NHANES is unique in that it combines interviews, physical examinations, and laboratory evaluations to obtain a large amount of quantitative and qualitative data (25). Briefly, the survey examines approximately 5,000 persons each year from various counties across the U.S. who are divided into a total of 30 primary sampling units (PSUs), 15 of which are visited annually. All participants provided written informed consent in agreement with the Public Health Service Act prior to any data collection. Household questionnaires, telephone interviews, and examinations conducted by healthcare professionals and trained personnel were utilized to collect the data.

### 2.2 Study participants and exclusion criteria

The 2005–2006 and 2017–2018 NHANES cycles collected data on 19,602 individuals. In the analysis, the researchers excluded 5,536 individuals without VA data and all children under the age of 18. After excluding data from non-compliant respondents, we collected 5,351 respondents with both hepatitis B surface antigen (HBsAg) and VA (Supplementary Figure S1) and 242 respondents with both hepatitis C RNA (HCV-RNA) and VA (Supplementary Figure S2). Serum VA levels were compared between HBsAg or HCV-RNA serology-positive and -negative respondents to determine whether there was a difference in the serum VA concentration between HBsAg-and HCV-RNA-negative respondents. According to the World Health Organization, the blood vitamin A (retinol) concentration < 0.70 μmol/L is considered as VAD in children and adults. In practice, however, few adults reach this level of deficiency, so in this article we chose to use subclinical vitamin A deficiency (SVAD) as the study index, with a value of <1.12 μmol/L. The concentration of vitamin A detected in serum is the retinol concentration, which we express here as serum VA. Among these remaining individuals, those with valid serum VA concentrations and complete information on demographic, anthropometric, questionnaire, and laboratory variables, including sex, age, body mass index (BMI), the ratio of family income to poverty (PIR), ethnicity, education, cigarette use, alcohol use, diabetes status, high blood pressure status, daily dietary energy/vitamin A/retinol intake, blood counts, C-reactive protein (CRP), and liver and kidney function parameters, were included in the analysis.

### 2.3 Assessment of serum VA

The serum VA concentration is available in the datasets and is included as an exposure variable expressed as a continuous variable. NHANES-examined participants aged 6 years and older were eligible. In the analysis, serum specimens were processed, stored, and shipped to the Division of Laboratory Sciences, National Center for Environmental Health, Centers for Disease Control and Prevention, Atlanta, GA, for analysis. The vials were frozen at −30°C until they were shipped to the National Center for Environmental Health for testing. The serum VA concentration was measured using modified high-performance liquid chromatography with a photodiode array detection method. The NHANES quality assurance and quality control protocols met the 1988 Clinical Laboratory Improvement Act mandates.

### 2.4 Assessment of hepatitis serology

HBsAg was tested by using the VITROS HBsAg test, the VITROS HBsAg kit on the VITROS ECi/ECiQ Immunodiagnostic System and VITROS 3600 Immunodiagnostic System, and the VITROS Immunodiagnostic Product HBsAg Calibrator. The principle of the test is based on an immunoassay technique consisting of a simultaneous reaction of HBsAg in the sample with the mouse monoclonal anti-HBs antibody coated on the wells and the mouse monoclonal anti-HBs antibody labeled with horseradish peroxidase (HRP) in the conjugate. The unbound conjugate is removed by washing and reagents consisting of luminescent substrates (luminescent phenol derivatives and peroxides) and electron transfer agents are added to the wells. The HRP in the bound conjugate catalyzes the oxidation of the luminescent phenol derivative to generate light. The electron transfer agent increases the intensity and prolongs the duration of the light. The light signal is read out with the VITROS ECi/ECiQ immunodiagnostic system. Ultimately, the amount of bound HRP conjugate indicates the amount of HBsAg in the sample (26). Hepatitis C ribonucleic acid was tested by using the COBAS Amplicon HCV Monitor test. The COBAS Amplicon HCV Monitor version 2 0 (v2.0) is an *in vitro* nucleic acid amplification test for the quantification of HCV-RNA in human serum or plasma on a COBAS Amplicon analyzer (27).

### 2.5 Defining demographic variables

Covariates related to serum VA, as well as potential confounders, were included based on the results of the literature searches. Participants were classified according to age, PIR, BMI, highest level of education, cigarette use, alcohol use, high blood pressure status, and diabetes status. Age was categorized according to international standards. PIR and BMI were classified by the triple fraction method. The participants’ levels of education were based on their responses during the home interview. High blood pressure status and diabetes status were recorded as yes or no response from the home interview. Diabetes status was defined as a fasting serum glucose >126 mg/dL, having answered “yes” to taking diabetic medications, or having been diagnosed by a physician with diabetes. A high blood pressure status was defined by whether a person was told that he/she has high blood pressure by a physician, if the blood pressure was >140/90 mmHg, and/or if that person is currently taking antihypertensive drugs. Alcohol use was divided into three categories: “non-drinker,” “moderate drinker,” and “heavy drinker.” Cigarette use was divided into two categories: “<100″ and “≥100″.

### 2.6 Statistics

Continuous variables are reported as the mean ± standard deviation (SD) or median with interquartile range (IQR). Categorical variables are presented as counts and percentages. Continuous variables were compared between groups using Mann–Whitney *U-*test or Student’s *t-*test, as appropriate. Categorical variables were compared using the chi-square test or Fisher’s exact test. A *p* < 0.05 was considered to indicate statistical significance. All the statistical analyses were performed by using STATA version 17. The association between vitamin A levels and the incidence of hepatitis was first estimated using a univariate logistic regression model or linear regression model. Multivariate logistic regression models or linear regression models were used to further evaluate the associations between them.

Logistic regression analysis using the Enter method was performed to assess the association between VAD and HBsAg/HCV-RNA in the different models. In the adjusted models, Model 1 made no adjustments, Model 2 adjusted for demographic variables, and Model 3 adjusted for variables with Model 2 and *P* < 0.05 according to Student’s *t*-test and Mann–Whitney *U-*test. Linear regression analyses were also conducted in the same way as those used in the logistic regression models.

## 3 Results

### 3.1 Study population

#### 3.1.1 Participants with HBsAg/HCV-RNA

The characteristics of the study subjects were divided according to HBsAg/HCV-RNA and VA levels and are presented in Table 1. The overall weighted incidence of hepatitis C was 43.0% (104/242), with males and females accounting for 69.2 and 30.8%, respectively, of the total HCV-RNA (+) population (*p* = 0.007). Notably, the VA level was significantly lower in HBsAg (+)/HCV-RNA (+) patients than in serologically virus-negative patients [1.63 (1.33 ~ 2.10) μg/L vs. 1.92 (1.57 ~ 2.34) μg/L, *P* < 0.001; 1.54 (1.25 ~ 1.83) μg/L vs. 1.78 (1.46 ~ 2.26) μg/L, *P* < 0.001]. The serum ALB concentration was significantly lower in HBsAg (+)/HCV-RNA (+) patients than in serologically virus-negative patients [(3.99 ± 0.36) g/dL vs. (4.14 ± 0.41) g/dL, *P* < 0.001; (3.84 ± 0.43) g/dL vs. (4.06 ± 0.36) g/dL, *P* < 0.001].

**Table 1:** Weighted characteristics of the study participants (US adults), according to HBsAg or HCV-RNA seropositivity.

Hepatitis B and C related indicators						
	HBsAg(+)	HBsAg(−)	*p*-value	HCV-RNA(+)	HCV-RNA(−)	*p*-value
No.	52	5,299		104	138	
Sex (%)			*P* = 0.658^b^			*P* = 0.007^b^
Male	27(51.9%)	2,588(48.8%)		72(69.2%)	72(52.2%)	
Female	25(48.1%)	2,711(51.2%)		32(30.8%)	66(47.8%)	
Age (year)	57(40.3–64)	45(28–63)	*P* = 0.070^a^	54(46–60)	58(45–66)	*P* = 0.019^a^
Age group (%)			*P* = 0.007^b^			*P* = 0.095^b^
<45	16(30.8%)	2,636(49.7%)		22(21.2%)	32(23.2%)	
(45, 60)	19(36.5%)	1,170(22.1%)		60(57.7%)	45(32.6%)	
>60	17(32.7%)	1,493(28.2%)		22(21.2%)	61(44.2%)	
Race (%)			*P* < 0.001^c^			*P* = 0.116^c^
Mexican American	1(1.9%)	1,078(20.3%)		8(7.7%)	18(13.0%)	
Other Hispanic	4(7.7%)	177(3.3%)		4(3.8%)	11(8.0%)	
Non-Hispanic White	6(11.5%)	2,411(45.5%)		43(41.3%)	56(40.6%)	
Non-Hispanic Black	19(36.5%)	1,289(24.3%)		43(41.3%)	39(28.3%)	
Other race	22(42.3%)	344(6.5%)		6(5.8%)	14(10.1%)	
PIR	1.68(1.06–4.86)	2.25(1.16–4.08)	*P* = 0.224^a^	1.32(0.75–2.41)	1.69(0.95–3.46)	*P* = 0.006^a^
Education (%)			*P* = 0.143^c^			*P* = 0.143^c^
< High school	13(25%)	1,325(25%)		30(28.8%)	27(19.6%)	
High school graduate/some college	24(46.2%)	2,991(56.4%)		66(63.5%)	93(67.4%)	
College graduate or above	15(28.8%)	983(18.6%)		8(7.7%)	18(13.0%)	
BMI (kg/m^−2^)	27.68 ± 6.57	28.52 ± 6.80	*P* = 0.203^d^	27.58 ± 6.24	28.76 ± 7.30	*P* = 0.316^d^
BMI group (%)			*P* = 0.282^b^			*P* = 0.491^b^
<25	18(34.6%)	1,710(32.3%)		35(33.7%)	48(34.8%)	
(25, 30)	21(40.4%)	1,751(33.0%)		36(34.6%)	39(28.3%)	
>30	12(23.1%)	1,760(33.2%)		28(26.9%)	45(32.6%)	
Energy intake (kcal)	1721(1368–2,352)	2,000(1456–2,665)	*P* = 0.053^a^	2,346(1539–3223)	1,998(1176–2743)	*P* = 0.055^a^
Vitamin A, RAE (mcg)	316(213–620)	455(24–768)	*P* = 0.089^a^	444(209–741)	420(213–764)	*P* = 0.773^a^
Retinol (mcg)	201(73–331)	311(156–550)	*P* = 0.002^a^	324(134–563)	296(117–556)	*P* = 0.512^a^
High blood pressure (%)			*P* = 0.091^b^			*P* = 0.037^b^
Yes	18(34.6%)	1,604(30.3%)		38(36.5%)	69(50%)	
No	33(63.5%)	3,682(69.5%)		66(63.5%)	69(50%)	
Refusal	1(1.9%)	13(0.2%)		0(0%)	0(0%)	
Diabetes (%)			*P* = 0.010^b^			*P* = 0.057^b^
Yes	12(23.1%)	508(9.6%)		12(11.5%)	26(18.8%)	
No	40(76.9%)	4,687(88.5%)		92(88.5%)	108(78.3%)	
Refusal	0(0%)	104(2.0%)		0(0%)	4(2.9%)	
Alcohol use (%)			*P* = 0.864^b^			*P* = 0.288^b^
Never or rarely	8(26.7%)	958(25.0%)		21(23.3%)	35(31.3%)	
Sometimes	17(56.7%)	2,085(54.4%)		49(54.4%)	60(53.6%)	
Often	5(16.7%)	792(20.7%)		20(22.2%)	17(15.2%)	
Cigarette use (%)			*P* = 0.564^b^			*P* < 0.001^b^
<100	29(56.9%)	2,529(52.8%)		11(10.7%)	51(38.1%)	
≥100	22(43.1%)	2,260(47.2%)		92(89.3%)	83(61.9%)	
Drugs						
Serum VA (μmol/L)	1.63(1.33–2.01)	1.92(1.57–2.34)	*P* < 0.001^a^	1.54(1.25–1.83)	1.78(1.46–2.26)	*P* < 0.001^a^
SVAD			*P* = 0.003^b^			*P* = 0.043^b^
Yes	9(17.3%)	365(6.9%)		16(15.4%)	10(7.2%)	
No	43(82.7%)	4,934(93.1%)		88(84.6%)	128(92.8%)	
Hemoglobin (g/dL)	14.10 ± 1.28	14.19 ± 1.59	*P* = 0.141^d^	14.34 ± 1.89	13.98 ± 1.54	*P* = 0.102^d^
Platelet count (1,000 cells/μL)	236.50(176.00–271.75)	266.00(225.00–314.00)	*P* < 0.001^a^	229.00(181.00–284.75)	241.00(195.75–297.75)	*P* = 0.191^a^
White blood cell count (1,000 cells/μL)	6.40(4.93–8.35)	7.10(5.90–8.60)	*P* = 0.058^a^	6.80(5.70–8.46)	7.10(5.90–8.40)	*P* = 0.549^a^
Segmented neutrophils percent (%)	55.65 ± 10.62	58.32 ± 10.16	*P* = 0.869^d^	54.33 ± 11.17	58.56 ± 10.55	*P* = 0.124^d^
Lymphocyte percent (%)	34.10(27.00–38.90)	29.60(24.00–35.8)	*P* = 0.012^a^	31.55(25.45–39.20)	29.65(23.58–35.80)	*P* = 0.047^a^
C-reactive protein (mg/L)	0.73(0.28–1.99)	0.24(0.08–0.62)	*P* < 0.001^a^	0.41(0.09–1.79)	1.33(0.39–3.57)	*P* < 0.001^a^
Albumin (g/dL)	3.99 ± 0.36	4.14 ± 0.41	*P* < 0.001^d^	3.84 ± 0.43	4.06 ± 0.36	*P* < 0.001^d^
Total bilirubin (μmol/L)	10.26(5.13–13.68)	10.26(8.55–13.68)	*P* = 0.142^a^	10.26(6.84–13.68)	8.55(5.13–11.97)	*P* = 0.004^a^
Alanine aminotransferase ALT (IU/L)	24(18.00–31.00)	20(16.00–28.00)	*P* = 0.032^a^	41(28.00–65.75)	19(12.00–26.00)	*P* < 0.001^a^
Aspartate aminotransferase AST (IU/L)	24(20.00–30.00)	23(19.00–27.00)	*P* = 0.222^a^	40(29.00–71.75)	20(18.00–25.00)	*P* < 0.001^a^
Alkaline phosphatase (ALP) (IU/L)	74.00(62.00–90.00)	69.00(56.00–85.00)	*P* = 0.044^a^	73.50(62.25–95.50)	79.00(62.00–94.50)	*P* = 0.528^a^
Creatinine (mg/dL)	0.88(0.71–1.01)	0.90(0.73–1.00)	*P* = 0.643^a^	0.90(0.80–1.00)	0.90(0.75–1.10)	*P* = 0.428^a^
Blood urea nitrogen (mg/dL)	13(10.00–17.00)	12(9.00–15.00)	*P* = 0.025^a^	13(9.00–16.00)	14(10.50–17.50)	*P* = 0.039^a^
Uric acid (μmol/L)	321.20(279.60–380.70)	309.30(255.80–374.70)	*P* = 0.332^a^	339.00(281.08–397.08)	327.10(249.80–389.60)	*P* = 0.102^a^
Total calcium (mg/dL)	9.39 ± 0.35	9.46 ± 0.38	*P* = 0.083^d^	9.24 ± 0.44	9.39 ± 0.45	*P* = 0.026^d^
Cholesterol (mg/dL)	190.33 ± 47.23	196.94 ± 43.91	*P* = 0.397^d^	177.35 ± 38.24	189.31 ± 42.75	*P* = 0.036^d^
HDL-cholesterol (mmol/L)	1.40(1.06–1.76)	1.34(1.11–1.66)	*P* = 0.837^a^	1.36(1.10–1.70)	1.29(1.03–1.59)	*P* = 0.363^a^
LDL-cholesterol (mmol/L)	2.91(2.13–3.84)	2.85(2.22–3.49)	*P* = 0.901^a^	2.53(2.19–3.28)	2.87(2.41–3.49)	*P* = 0.169^a^
Glycohemoglobin (%)	6.16 ± 1.51	5.54 ± 1.01	*P* < 0.001^d^	5.61 ± 1.14	5.93 ± 1.26	*P* = 0.173^d^

Data are presented as means (SD), median (quartile 1, quartile 3) or number (percentage). SVAD, Subclinical vitamin A deficiency; VA, vitamin A; PIR, The ratio of family income to poverty; BMI, Body mass index; HBsAg, Hepatitis B surface antigen; HCV-RNA, Hepatitis C RNA. ^a^Mann-Whitney *U*-test. ^b^Chi-squared test. ^c^Fisher exact test. ^d^Student’s *t*-test.

#### 3.1.2 Participants with SVAD

The characteristics of the study subjects with SVAD (VA < 1.12 μmol/L) are shown in Supplementary Tables S1, S2. Compare to the normal vitamin A (VAN) group, SVAD was lower in the daily dietary intake, including energy intake (kcal) [1800 (1,340 ~ 2,438) vs. 2013(1,464 ~ 2,681), *P* < 0.001], vitamin A (RAE) (mcg) [342(159 ~ 594) vs. 464(253 ~ 776), *P* < 0.001], and retinol (mcg) [223(92 ~ 450) vs. 314(160 ~ 554), *P* < 0.001] among participants with hepatitis B. The overall weighted prevalence of hepatitis B was 2.4% in SVAD group higher than 0.9% in VAN group (Supplementary Figure S3A). The results also showed that SVAD was more common in women than in men [78.9% (295/374) vs. 21.1% (79/374), *P* < 0.001] among respondents with HBsAg (Supplementary Figure S4). Similar results were obtained for daily dietary intake in the hepatitis C population, but the difference was not significant. The overall weighted incidence of hepatitis C was 61.5% in the SVAD group, which was greater than the 40.7% in the VAN group (Supplementary Figure S3B).

### 3.2 Associations between VA and the prevalence of HBsAg (+)/HCV-RNA (+)

#### 3.2.1 SVAD and viral hepatitis

**Figure 1:**
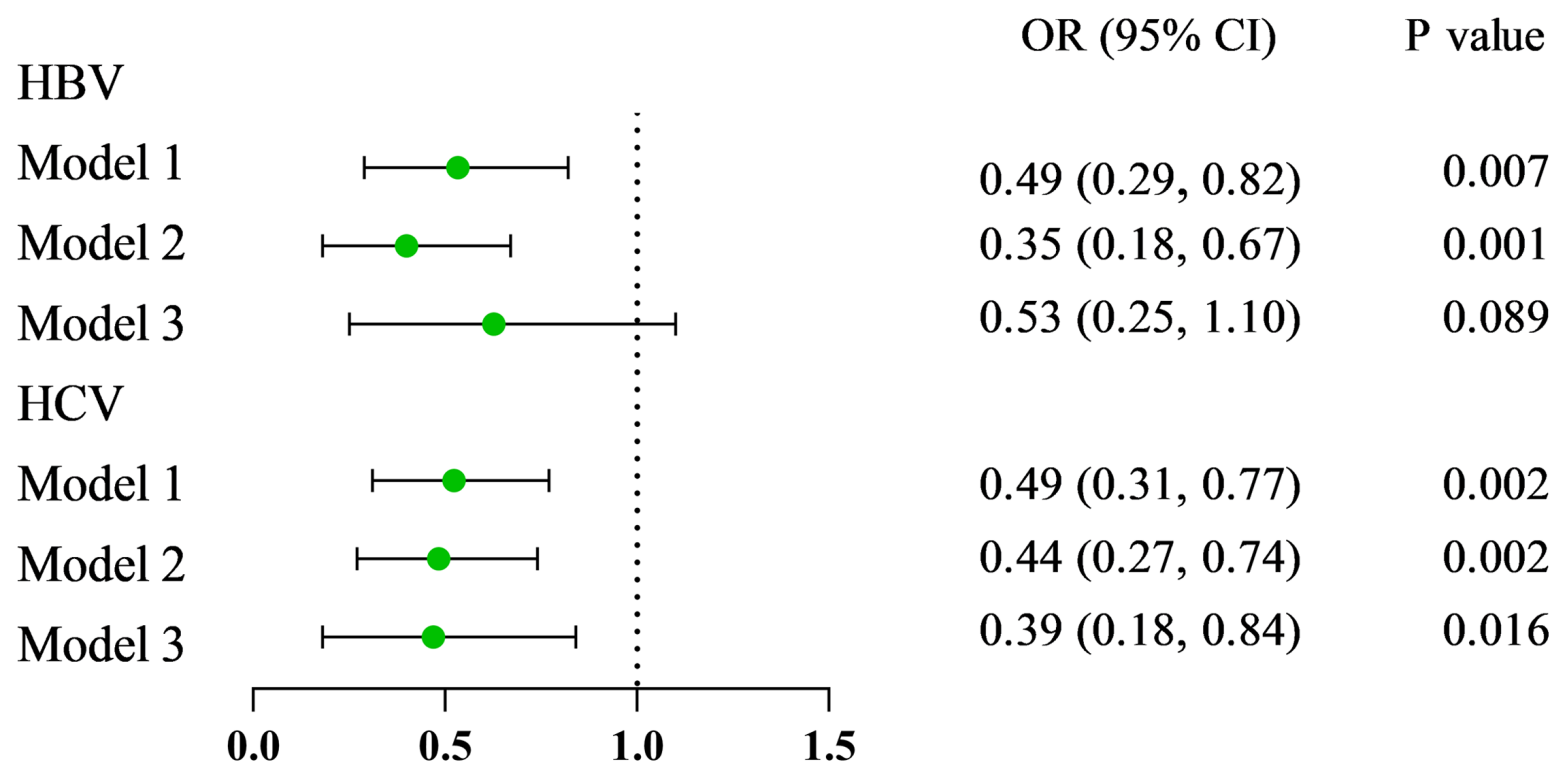
The forest plots of the relationship between SVAD and viral hepatitis.

As presented in Figure 1, VA was negatively related to the incidence of HBsAg (+)/HCV-RNA (+). The odds ratios (ORs) were 0.49 (95% CI = 0.29 ~ 0.82), 0.35 (95% CI = 0.18 ~ 0.67), and 0.53 (95% CI = 0.25 ~ 1.10) in Model 1, Model 2, and Model 3, respectively, among respondents with HBsAg (+) (Table 2). Moreover, among respondents with HCV-related RNA (+), the ORs were 0.49 (95% CI = 0.31 ~ 0.77), 0.44 (95% CI = 0.27 ~ 0.74), and 0.39 (95% CI = 0.18 ~ 0.84) in Model 1, Model 2, and Model 3, respectively (Table 3).

**Table 2:** Associations between hepatitis B among participants with SVAD.

Variable	Hepatitis B	
	OR (95% CI)	*p*-value
Serum VA		
Model 1	0.49 (0.29, 0.82)	0.007
Model 2	0.35 (0.18, 0.67)	0.001
Model 3	0.53 (0.25, 1.10)	0.089

Model 1: Non-adjusted. Model 2: Adjustment of sociodemographic variables including sex, race, age and BMI + socioeconomic status. Variables including PIR and education level. Model 3: Adjustment of covariates in model 2 and important variables including Retinol intake, PLT, Lymphocyte percent, CRP, ALB, ALT, ALP, BUN, diabetes, and Glycohemoglobin. OR, Odds ratio; CI, 95% confidence interval; PIR, The ratio of family income to poverty; BMI, Body mass index; PLT, Platelet count; CRP, C-reactive protein; ALB, Albumin; ALT, Alanine aminotransferase; ALP, Alkaline phosphatase; BUN, Blood urea nitrogen; SVAD, Subclinical vitamin A deficiency.

**Table 3:** Associations between hepatitis C among participants with SVAD.

Variable	Hepatitis C	
	OR (95% CI)	*p*-value
Serum VA		
Model 1	0.49 (0.31, 0.77)	0.002
Model 2	0.44 (0.27, 0.74)	0.002
Model 3	0.39 (0.18, 0.84)	0.016

Model 1: Non-adjusted. Model 2: Adjustment of sociodemographic variables including sex, race, age and BMI + socioeconomic status. Variables including PIR, and education level. Model 3: Adjustment of covariates in model 2 and important variables including Lymphocyte percent, CRP, ALB, ALT, AST, BUN, Total calcium, Cholesterol, and high blood pressure. OR, Odds ratio; CI, 95% confidence interval; PIR, The ratio of family income to poverty; BMI, Body mass index; CRP, C-reactive protein; ALB, Albumin; ALT, Alanine aminotransferase; AST, Aspartate aminotransferase; BUN, Blood urea nitrogen; SVAD, Subclinical vitamin A deficiency.

#### 3.2.2 Viral hepatitis and VA levels

**Figure 2:**
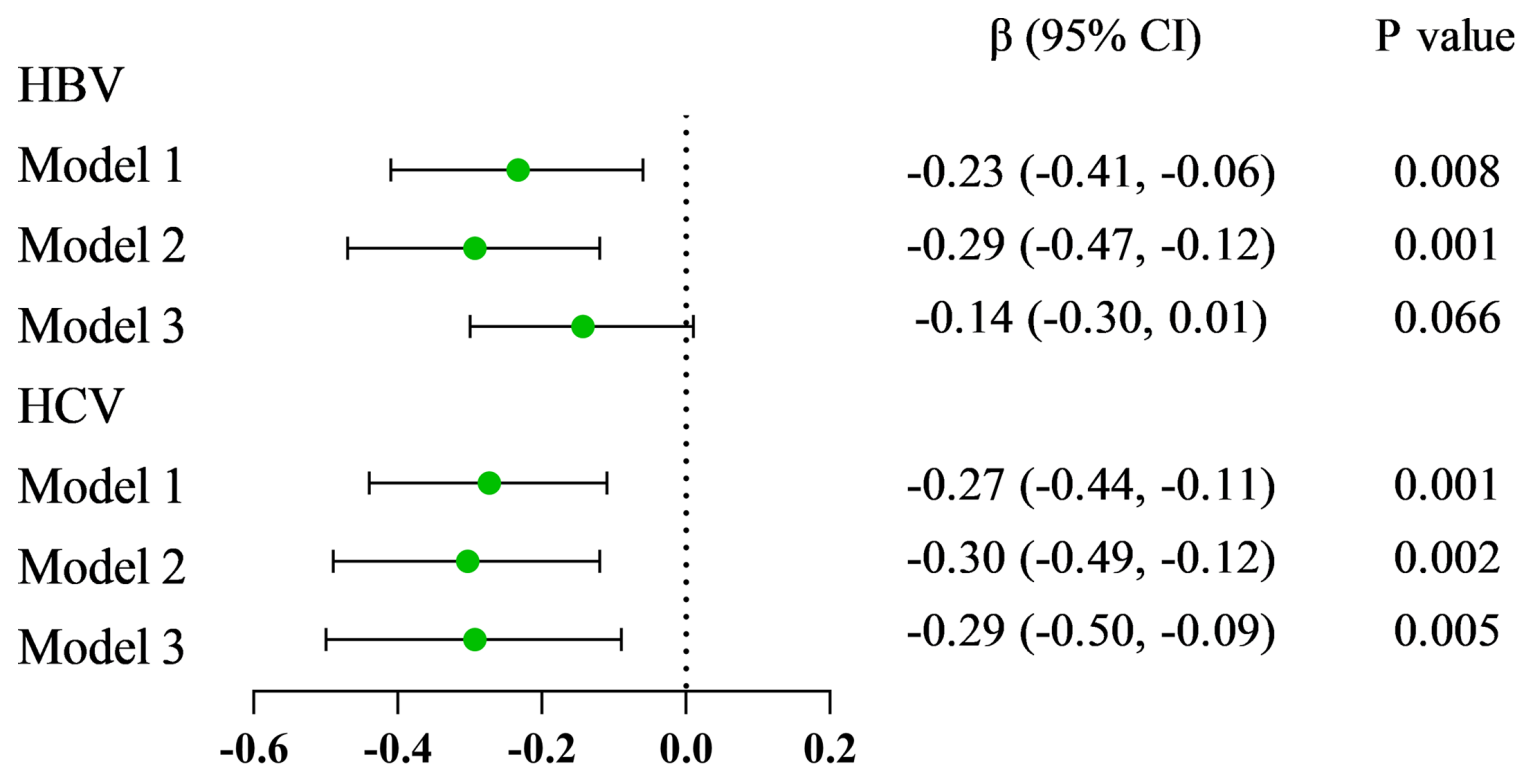
The forest plots of the relationship between viral hepatitis and VA levels.

The results from linear regression models are shown in Figure 2. An inverse association between serum VA as a continuous factor and HBsAg was observed (*β* = −0.23, 95% CI = −0.41 to −0.06, *p* = 0.008; *β* = −0.29, 95% CI = −0.47 to −0.12, *p* = 0.001; *β* = −0.14, 95% CI = −0.30 ~ 0.01, *p* = 0.066) (Table 4). Moreover, among the respondents with HCV-related RNA, there was also a negative correlation (*β* = −0.27, 95% CI = −0.44 to −0.11; *P* = 0.001; *β* = −0.30, 95% CI = −0.49 to −0.12; *p* = 0.002; *β* = −0.29, 95% CI = −0.50 to −0.09; *p* = 0.005) (Table 5).

**Table 4:** Associations between serum VA among participants with hepatitis B.

Variable	HBsAg (−)	HBsAg (+)	*p*-value
Serum VA			
Model 1 *β* (95% CI)	0	−0.23 (−0.41, −0.06)	0.008
Model 2 *β* (95% CI)	0	−0.29 (−0.47, −0.12)	0.001
Model 3 *β* (95% CI)	0	−0.14 (−0.30, 0.01)	0.066

Model 1: Non-adjusted. Model 2: Adjustment of sociodemographic variables including sex, race, age and BMI + socioeconomic status. Variables including PIR and education level. Model 3: Adjustment of covariates in model 2 and important variables including Energy intake, REA, Retinol intake, Cigarette use, Segmented neutrophils percent, Lymphocyte percent, CRP, ALB, TB, ALT, AST, ALP, Creatinine, BUN, UA, Cholesterol, HDL-Cholesterol, and high blood pressure. HBsAg, hepatitis B surface antigen; CI, 95% confidence interval; PIR, The ratio of family income to poverty; BMI, Body mass index; REA, Vitamin A intake; CRP, C-reactive protein; ALB, Albumin; TB, Total bilirubin; ALT, Alanine aminotransferase; AST, Aspartate aminotransferase; ALP, Alkaline phosphatase; Cr, Creatinine; BUN, Blood urea nitrogen; UA, Uric acid; VA, Vitamin A.

**Table 5:** Associations between serum VA among participants with hepatitis C.

Variable	HCV-RNA (−)	HCV-RNA (+)	*p*-value
Serum VA			
Model 1 *β* (95% CI)	0	−0.27 (−0.44, −0.11)	0.001
Model 2 *β* (95% CI)	0	−0.30 (−0.49, −0.12)	0.002
Model 3 *β* (95% CI)	0	−0.29 (−0.50, −0.09)	0.005

Model 1: Non-adjusted. Model 2: Adjustment of sociodemographic variables including sex, race, age and BMI + socioeconomic status. Variables including PIR and education level. Model 3: Adjustment of covariates in model 2 and important variables including WBC, CRP, AST, and BUN. HCV-RNA, hepatitis C RNA; CI, 95% confidence interval; PIR, The ratio of family income to poverty; BMI, body mass index; WBC, White blood cell count; CRP, C-reactive protein; AST, Aspartate aminotransferase; BUN, Blood urea nitrogen; VA, vitamin A.

## 4 Discussion

VA is an immunomodulatory substance, and studies have confirmed its anti-infective effects; deficiency of this substance may lead to an imbalanced inflammatory response and impaired immune function. HBV and HCV infections are the major causes of acute and chronic hepatitis worldwide and can lead to liver cirrhosis and hepatocellular carcinoma (HCC) (28). Our study showed that the incidence of SVAD is greater in patients with HBV or HCV infection and that serum virus-positive patients have lower levels of VA than serum virus-negative patients.

Several studies have shown that endogenous VA is involved in the body’s immunity, and a large amount of loss leads to further aggravation of the disease (29–31). This may create a vicious cycle between viral hepatitis and VA. Moreover, the latest studies have reported that exogenous supplementation of VA may be a target for improving immunity and fighting viral infections (32). However, the results of our data analysis do not explain whether VA deficiency leads to an increase in the rate of viral infections or whether viral infections lead to a decrease in the body’s VA levels. This supposition needs to be confirmed by further clinical studies.

In this cross-sectional study of U.S. adults, serum VA was inversely associated with serum viral positivity, and subjects with concurrent SVAD were more likely to be virus positive. Hepatitis B has been reported to affect VA storage in several ways (33). With impaired liver function after hepatitis, the storage of retinyl esters may be reduced, as may the ability of retinyl esters to mobilize hydrolysis and dehydroconversion to retinol, which may explain the low serum retinol concentration in patients with impaired liver function (34). Interestingly, our study showed that the daily intake of VA and retinol in the included population was less than the amount recommended by the World Health Organization and that VA-deficient subjects consumed even less VA (Tables 2, 3). Therefore, there may be a correlation between serum VA levels and viral positivity and between the serum VA concentration and viral intake, and additional detailed details need to be verified in additional clinical trials.

VA and its derivatives play crucial roles in controlling cell growth and differentiation, inhibiting liver cell transformation, inhibiting the proliferation of liver tumor cells, inhibiting the production of proinflammatory cytokines in macrophages, reducing inflammatory reactions, and protecting organisms from oxidative stress (OS) (35, 36). Notably, studies of VA and viral infections, including early childhood hand, foot and mouth disease (3), measles virus (24), and hepatitis C virus, are not uncommon. Furthermore, our previous research has shown that the prevalence of VAD is high in children with sepsis and that VAD may be a marker of mortality in critically ill children with sepsis (4). A wealth of data indicates that patients with these viral infections exhibit low VA levels, which may be related to an immunocompromised status and reduced VA stores following liver damage. However, additional in-depth basic research is needed to clarify the specific underlying mechanisms involved. Overall, this approach can prompt clinicians to pay attention to patients’ VA levels when encountering viral infections or severe infections to provide new ideas for later treatment protocols.

For the past few years, several studies have shown that transretinoic acid can be used to treat hepatitis C. Vitamin A upregulates type 1 IFN receptor expression and enhances the anti-HCV replication effects of IFN-α (37). In a cohort of previously unresponsive patients with chronic HCV infection, all-trans retinoic acid (ATRA) had direct antiviral effects and strong additive or synergistic effects with polyethylene glycol IFN (38). However, little research has been conducted on VA for the treatment of hepatitis B. In contrast, there are several reported side effects of VA on the liver in previous articles, revealing that VA is a proven hepatotoxic agent that can cause conditions ranging from acute hepatitis to cirrhosis, particularly short-term high dose use of VA (39). We are currently conducting trials on the efficacy and safety of VA-assisted therapy for children with sepsis to analyze whether VA supplementation improves the clinical prognosis in patients with sepsis. VA is necessary for an adequate immune response to infection. Several studies have shown that VAD leads to increased systemic inflammation (40, 41). The decreased VA levels under inflammatory conditions may be related to low levels of retinol binding protein (RBP) and leakage of RBP into the extravascular space (42). In addition, some studies have reported that the plasma retinol concentration decreases during inflammation and sepsis, and retinol is secreted into the urine (43). In western Kenya, there was a significant positive association with infection among VA-deficient (RB*P* < 0.83 μmol/L) infants (44). Infants with iron deficiency were less likely to have infection based on the CRP level, while infants with VAD were five times more likely to have infection. The decrease in plasma AST activity in patients with urolithiasis might be attributed to supplementation with the vitamin ADEK. Patients with fat malabsorption may benefit from the supplementation of the fat-soluble vitamin ADEK (45). Conversely, it has also been found that serum VA was positively associated with the degree of non-alcoholic fatty liver disease (NAFLD), especially in the non-obese population (46). This finding is contrary to our findings. It also suggests us that various liver diseases, hepatitis and liver injury (trauma) may affect serum VA concentration. This needs to be confirmed by more prospective studies and explored at the basic research level.

## 5 Limitations

This study was subject to certain constraints. First, this was a cross-sectional investigation, precluding the determination of causality. Second, serum vitamin A (VA) levels were assessed only once, and any fluctuations within individuals might influence the observed association. However, the half-life of VA is extended in humans (approximately 140 days), and serum VA levels within the typical range are tightly controlled in individuals (47, 48). Third, despite meticulously considering various related covariates, we cannot exclude all potential residual confounding factors in observational studies. Finally, decreased serum vitamin A levels in hepatitis patients may be associated with decreased hepatic stellate cell retinyl ester storage as well as mobilization capacity and with general susceptibility of the population to hepatitis viruses, and whether decreased vitamin A levels result in a population with impaired immune function more susceptible to transition to chronic patients or carriers needs to be confirmed by more rigorous basic studies.

## 6 Conclusion

In this study, we found that patients with hepatitis viral infections had low levels of VA, that the rate of viral infection was high in patients with SVAD, and that the serum VA concentration was inversely associated with seropositivity for hepatitis B and C viruses. Our findings suggest that, in patients with hepatitis B or C viral infections, we should pay attention to their serologic VA levels, and timely supplementation may be twice as effective as conventional treatment.

## Data availability statement

The original contributions presented in the study are included in the article/Supplementary material, further inquiries can be directed to the corresponding author.

## Author contributions

ML: Conceptualization, Data curation, Formal analysis, Methodology, Project administration, Resources, Software, Visualization, Writing – original draft. JF: Data curation, Methodology, Software, Visualization, Writing – original draft. XZ: Methodology, Investigation, Validation, Writing – review & editing. QF: Investigation, Writing – review & editing, Project administration, Software. YJ: Writing – review & editing, Funding acquisition, Validation. SC: Funding acquisition, Validation, Writing – review & editing, Conceptualization, Resources, Supervision.

## Footnotes

^1^ https://wwwn.cdc.gov/Nchs/Nhanes/2005–2006/2017-2018/TST_H.htm
