# Supplementary material for: The association between serum vitamin A concentrations and virus hepatitis among U.S. adults from the NHANES database: a cross-sectional study
Source: Front Nutr. 2024 Aug 1;11:1387461. doi: 10.3389/fnut.2024.1387461 (PMC11324588; doi:10.3389/fnut.2024.1387461)
Supplement: Supplementary file 1 [file Data_Sheet_1.docx]

Supplementary Material

# Supplementary Figures and Tables

## Supplementary Figures


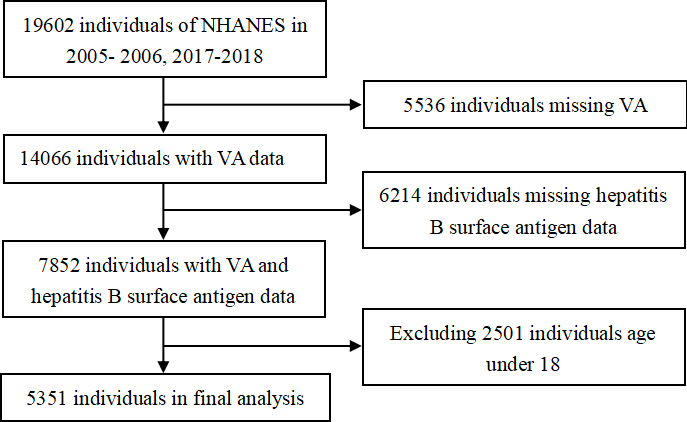


**Supplementary Figure 1.** Study flowchart of respondents associated with hepatitis B surface antigen. NHANES, National Health and Nutrition Examination Survey


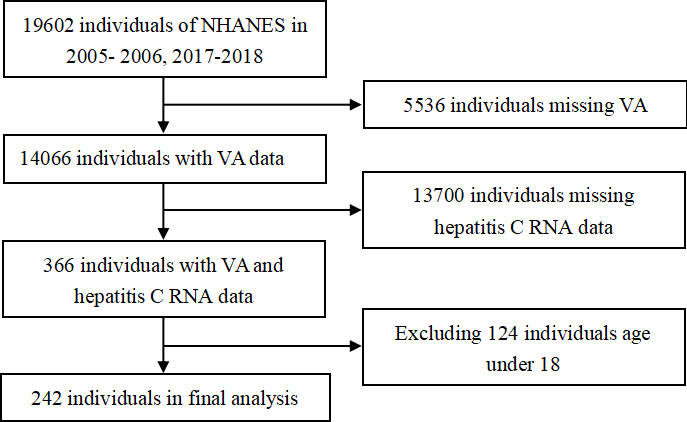


**Supplementary Figure 2.** Study flowchart of respondents associated with hepatitis C RNA. NHANES, National Health and Nutrition Examination Survey


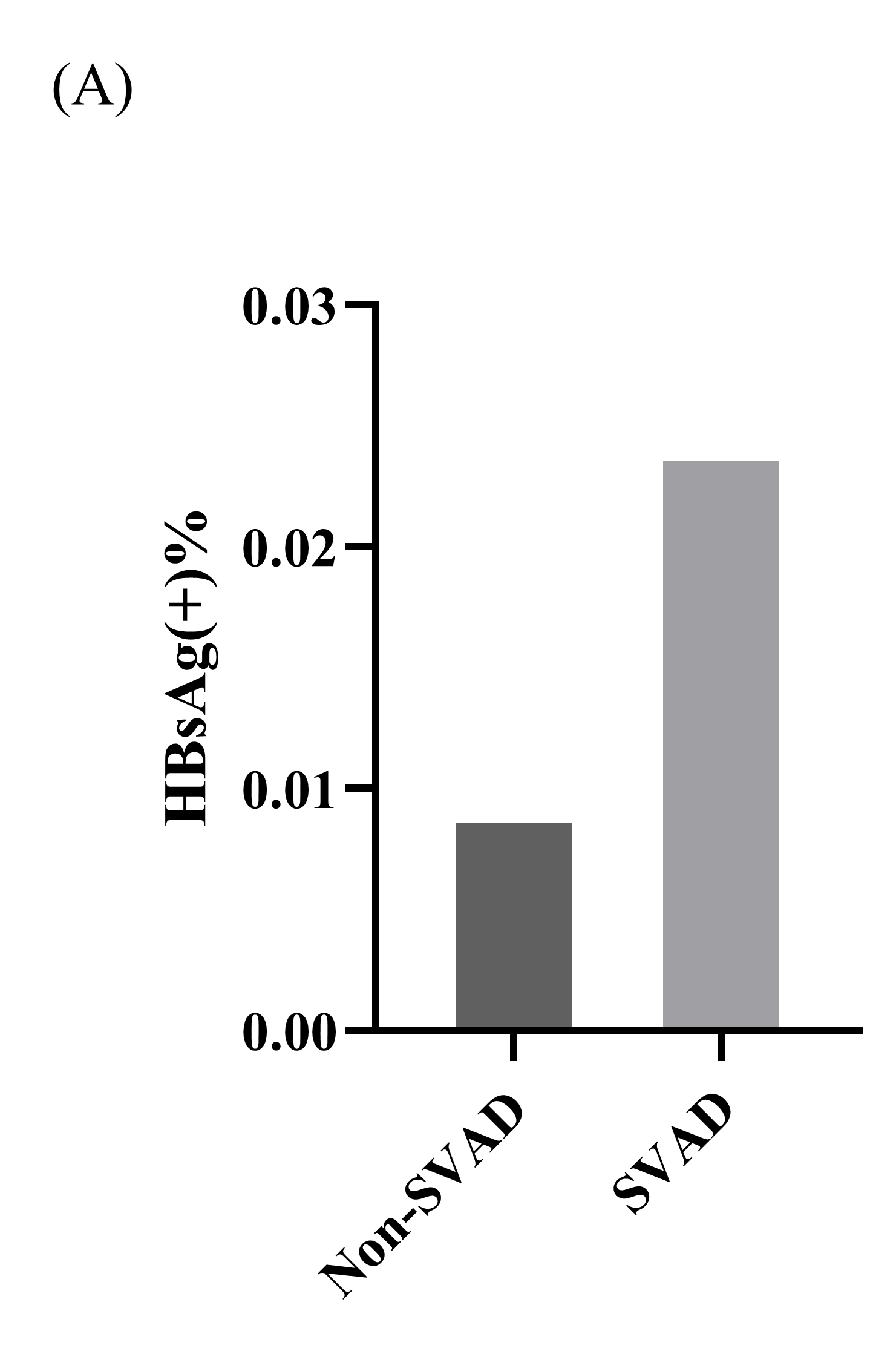

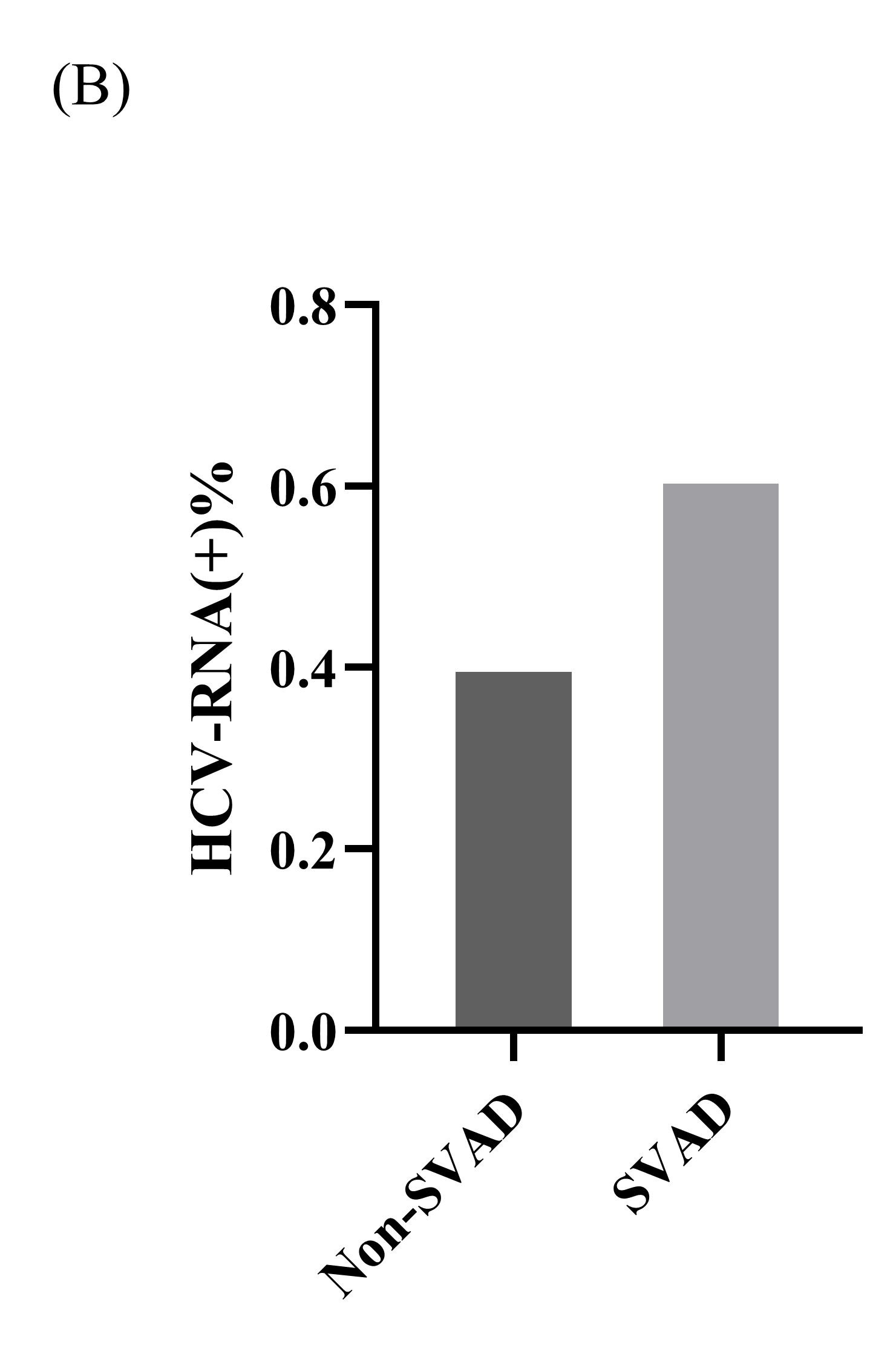


**Supplementary Figure 3.** A. Percentage of respondents with HBsAg (+) the virus in the entire group in both groups; B. Percentage of people with HCV-RNA (+) in the entire group in both groups.


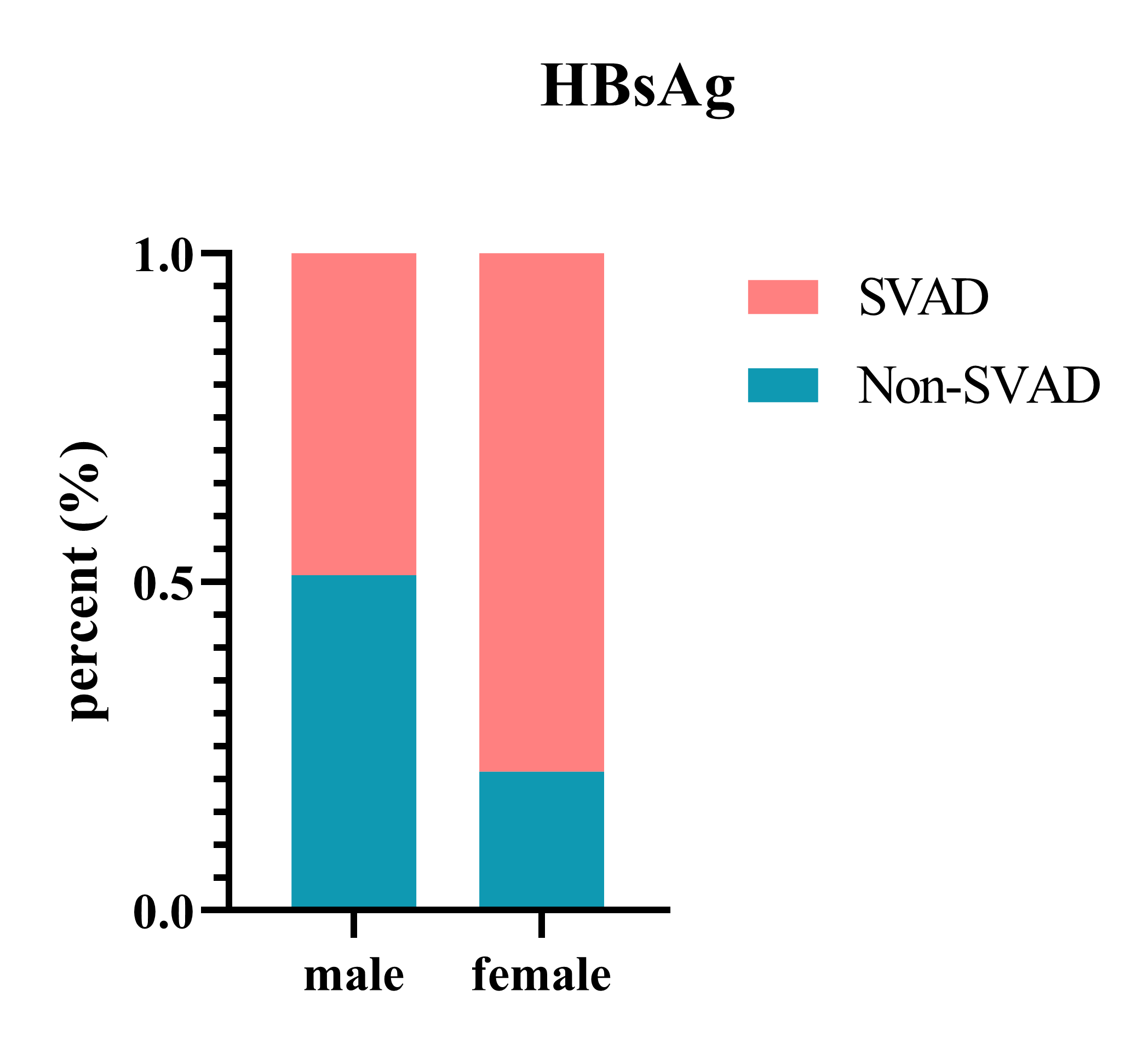


**Supplementary Figure 4.** Percentage of SVAD in the male and female subgroups of the population with data on HBsAg. SVAD Subclinical Vitamin A deficiency, HBsAg hepatitis B surface antigen

**Supplementary Table 1.** Associations between serum VA among participants with hepatitis B.

|  | VAN(≥1.12μmol/L)  (N=4977) | SVAD(<1.12μmol/L)  (N=374) | P-value |
| --- | --- | --- | --- |
| Serum VA | 1.97(1.65-2.38) | 1.09(0.97-1.15) | P<0.001^a^ |
| Sex(%) |  |  | P<0.001^b^ |
| male | 2536(51.0%) | 79(21.1%) |  |
| female | 2441(49.0%) | 295(78.9%) |  |
| Age(year) | 46(29-63) | 32.5(23-51) | P<0.001^a^ |
| Race(%) |  |  | P<0.001^c^ |
| Mexican American | 989(19.9%) | 90(24.1%) |  |
| Other hispanic | 166(3.3%) | 15(4.0%) |  |
| Non-hispanic white | 2330(46.8%) | 87(23.3%) |  |
| Non-hispanic black | 1152(23.1%) | 156(41.7%) |  |
| Other race | 340(6.8%) | 26(7.0%) |  |
| PIR | 2.27(1.19-4.17) | 1.68(0.82-3.12) | P<0.001^a^ |
| Education(%) |  |  | P=0.001^c^ |
| < high school | 1234(24.8%) | 104(27.8%) |  |
| High school graduate/some college | 2788(56.0%) | 227(60.7%) |  |
| College graduate or above | 955(19.2%) | 43(11.5%) |  |
| BMI(kg/m^−2^) | 27.35(23.90-31.67) | 28.43(23.50-33.23) | P=0.205^a^ |
| Energy intake(kcal) | 2013(1464-2681) | 1800(1340-2438) | P<0.001^a^ |
| Vitamin A, RAE (mcg) | 464(253-776) | 342(159-594) | P<0.001^a^ |
| Retinol (mcg) | 314(160-554) | 223(92-450) | P<0.001^a^ |
| high blood pressure(%) |  |  | P<0.001^b^ |
| Yes | 1540(30.9%) | 82(21.9%) |  |
| NO | 3425(68.8%) | 290(77.5%) |  |
| Refusal | 12(0.3%) | 2(0.6%) |  |
| diabetes(%) |  |  | P=0.619^b^ |
| Yes | 489(9.8%) | 31(8.3%) |  |
| NO | 4391(88.2%) | 336(89.8%) |  |
| Refusal | 97(2.0%) | 7(1.9%) |  |
| Alcohol use(%) |  |  | P=0.003^b^ |
| Never or rarely | 892(24.5%) | 74(33.3%) |  |
| Sometimes | 1985(54.5%) | 117(52.7%) |  |
| Often | 766(21.0%) | 31(14.0%) |  |
| Cigarette use(%) |  |  | P=0.001^b^ |
| <100 | 2360(52.2%) | 198(62.3%) |  |
| ≥100 | 2162(47.8%) | 120(37.7%) |  |
| Drugs |  |  |  |
| HBsAg, n, (%) |  |  | P=0.003^b^ |
| HBsAg(+) | 43(0.9%) | 9(2.4%) |  |
| HBsAg(–) | 4934(99.1%) | 365(97.6%) |  |
| Hemoglobin (g/dL) | 14.29±1.54 | 12.83±1.57 | P=0.531^d^ |
| Platelet count (1000 cells/uL) | 266.00(225.00-314.00) | 260.50(214.50-312.25) | P=0.052^a^ |
| White blood cell count (1000 cells/uL) | 7.10(5.90-8.60) | 7.25(5.80-9.00) | P=0.150^a^ |
| Segmented neutrophils percent (%) | 58.16±9.97 | 60.18±12.37 | P<0.001^d^ |
| Lymphocyte percent (%) | 29.80(24.10-35.80) | 27.10(20.30-35.60) | P<0.001^a^ |
| C-Reactive Protein (mg/L) | 0.23(0.08-0.58) | 0.53(0.18-1.38) | P<0.001^a^ |
| Albumin (g/dL) | 4.17±0.39 | 3.74±0.54 | P<0.001^d^ |
| Total Bilirubin (umol/L) | 11.97(8.55-13.68) | 9.41(6.84-11.97) | P<0.001^a^ |
| Alanine aminotransferase ALT (IU/L) | 21.00(16.00-28.00) | 16.00(13.00-23.00) | P<0.001^a^ |
| Aspartate aminotransferase AST (IU/L) | 23.00(20.00-28.00) | 20.00(18.00-26.00) | P<0.001^a^ |
| Alkaline Phosphatase (ALP) (IU/L) | 69.00(56.00-84.00) | 74.00(58.00-101.00) | P<0.001^a^ |
| Creatinine (mg/dL) | 0.90(0.80-1.10) | 0.70(0.60-0.80) | P<0.001^a^ |
| Blood urea nitrogen (mg/dL) | 12.00(9.00-15.00) | 8.00(6.00-11.00) | P<0.001^a^ |
| Uric acid (umol/L) | 255.80(214.10-309.30) | 315.2(261.7-374.7) | P<0.001^a^ |
| Total calcium (mg/dL) | 9.48±0.37 | 9.21±0.39 | P=0.062^d^ |
| Cholesterol (mg/dL) | 194.00(168.00-224.00) | 179.50(154.00-213.00) | P<0.001^a^ |
| HDL-Cholesterol (mmol/L) | 1.34(1.09-1.63) | 1.45(1.16-1.78) | P<0.001^a^ |
| LDL-Cholesterol (mmol/L) | 2.85(2.25-3.52) | 2.59(1.97-3.28) | P<0.001^a^ |
| Glycohemoglobin (%) | 5.56±1.01 | 5.50±1.15 | P=0.068^d^ |

Data are presented as means (SD), median (quartile 1, quartile 3) or number (percentage)

VAN normal vitamin A, SVAD Subclinical Vitamin A deficiency,VA vitamin A, PIR the ratio of family income to poverty, BMI body mass index, HBsAg hepatitis B surface antigen

^a^Mann-Whitney U test

^b^Chi-squared test

^c^Fisher exact test

^d^Student’s t test

**Supplementary Table 2.** Associations between serum VA among participants with hepatitis C.

|  | VAN(≥1.12μmol/L)  (N=215) | SVAD(<1.12μmol/L)  (N=26) | P-value |
| --- | --- | --- | --- |
| Serum VA | 1.72(1.45-2.13) | 0.85(0.76-1.01) | P<0.001^a^ |
| Sex(%) |  |  | P=0.842^b^ |
| male | 129(59.7%) | 15(57.7%) |  |
| female | 89(40.3%) | 11(42.3%) |  |
| Age(year) | 55.00(45.00-63.75) | 56.50(45.50-63.50) | P=0.931^a^ |
| Race(%) |  |  | P=0.480^c^ |
| Mexican American | 24(11.1%) | 2(7.7%) |  |
| Other hispanic | 14(6.5%) | 1 (3.8%) |  |
| Non-hispanic white | 91(42.1%) | 8(30.8%) |  |
| Non-hispanic black | 69(31.9%) | 13(50%) |  |
| Other race | 18(8.3%) | 2(7.7%) |  |
| PIR | 1.54(0.88-3.20) | 1.16(0.70-2.10) | P=0.156^a^ |
| Education(%) |  |  | P=0.380^c^ |
| < high school | 49(22.7%) | 8(30.8%) |  |
| High school graduate/some college | 142(65.7%) | 17(65.4%) |  |
| College graduate or above | 25(11.6%) | 1(3.8%) |  |
| BMI(kg/m^−2^) | 27.58(23.40-31.30) | 26.50(21.36-33.10) | P=0.537^a^ |
| Energy intake(kcal) | 2243(1374-2975) | 1734(918-2522) | P=0.138^a^ |
| Vitamin A intake, RAE (mcg) | 447(227-747) | 211(148-1002) | P=0.219^a^ |
| Retinol intake (mcg) | 325(137-556) | 152(55-582) | P=0.122^a^ |
| high blood pressure(%) |  |  | P=0.295^a^ |
| Yes | 93(43.1%) | 14(53.8%) |  |
| NO | 123(56.9%) | 12(46.2%) |  |
| diabetes(%) |  |  | P=0.631^b^ |
| Yes | 35(16.2%) | 3(11.5%) |  |
| NO | 177(81.9%) | 23(88.5%) |  |
| Refusal | 4(1.9%) | 0(0.0%) |  |
| Alcohol use(%) |  |  | P=0.598^b^ |
| Never or rarely | 50(27.5%) | 6(30.0%) |  |
| Sometimes | 97(53.3%) | 12(60.0%) |  |
| Often | 35(19.2%) | 2(10.0%) |  |
| Cigarette use(%) |  |  | P=0.459^b^ |
| <100 | 57(26.9%) | 5(20.0%) |  |
| ≥100 | 155(73.1%) | 20(80.0%) |  |
| Drugs |  |  |  |
| HCV-RNA, n, (%) |  |  | P=0.043^b^ |
| HCV-RNA(+) | 88(40.7%) | 16(61.5%) |  |
| HCV-RNA(–) | 128(59.3%) | 10(38.5%) |  |
| Hemoglobin (g/dL) | 14.21±1.62 | 13.48±2.21 | P=0.147^d^ |
| Platelet count (1000 cells/uL) | 234.00(192.75-294.00) | 228.00(143.25-271.50) | P=0.264^a^ |
| White blood cell count (1000 cells/uL) | 7.15(6.00-8.48) | 6.05(4.70-7.13) | P=0.009^a^ |
| Segmented neutrophils percent (%) | 58.70(50.05-64.70) | 53.55(42.73-63.63) | P=0.196^a^ |
| Lymphocyte percent (%) | 30.30(23.93-37.30) | 31.50(25.53-43.83) | P=0.287^a^ |
| C-Reactive Protein (mg/L) | 0.65(0.16-2.73) | 2.55(0.84-7.49) | P=0.001^a^ |
| Albumin (g/dL) | 3.99±0.38 | 3.68±0.47 | P=0.227^d^ |
| Total Bilirubin (umol/L) | 8.55(5.13-13.68) | 9.41(5.13-13.68) | P=0.773^a^ |
| Alanine aminotransferase ALT (IU/L) | 24.00(16.00-41.00) | 31.00(18.25-52.75) | P=0.214^a^ |
| Aspartate aminotransferase AST (IU/L) | 24.00(20.00-39.00) | 36.00(24.25-57.75) | P=0.031^a^ |
| Alkaline Phosphatase (ALP) (IU/L) | 78.00(62.00-93.00) | 84.00(62.50-118.00) | P=0.205^a^ |
| Creatinine (mg/dL) | 0.90(0.75-1.10) | 0.82 (0.79-0.98) | P=0.354^a^ |
| Blood urea nitrogen (mg/dL) | 14.00(10.00-17.00) | 11.00(6.75-15.75) | P=0.037^a^ |
| Uric acid (umol/L) | 333.10(267.70-398.50) | 318.20(257.25-379.18) | P=0.555^a^ |
| Total calcium (mg/dL) | 9.35±0.43 | 9.08±0.56 | P=0.216^d^ |
| Cholesterol (mg/dL) | 186.19±40.76 | 167.27±41.87 | P=0.589^d^ |
| HDL-Cholesterol (mmol/L) | 1.32(1.06-1.63) | 1.33(1.13-1.64) | P=0.981^a^ |
| LDL-Cholesterol (mmol/L) | 2.87(2.33-3.47) | 2.28(1.73-3.25) | P=0.108^a^ |
| Glycohemoglobin (%) | 5.79±1.21 | 5.80±1.30 | P=0.501^d^ |

Data are presented as means (SD), median (quartile 1, quartile 3) or number (percentage)

VAN normal vitamin A, SVAD Subclinical Vitamin A deficiency,VA vitamin A, PIR the ratio of family income to poverty, BMI body mass index, HCV-RNA hepatitis C RNA

^a^Mann-Whitney U test

^b^Chi-squared test

^c^Fisher exact test

^d^Student’s t test
